# Estimation of compressive strength of ultra-high performance lightweight concrete (UHPLC) using neural network

**DOI:** 10.1371/journal.pone.0326652

**Published:** 2025-07-07

**Authors:** Yan Zhao, Ziyan Huang, Huilong Zhao, Zhen Xu, Wei Chang, Bai Liu

**Affiliations:** 1 School of Civil Engineering and Architecture, Wuyi University, Wuyishan City, Fujian Province, China; 2 Nanping Highway Development Center, Nanping City, Fujian Province, China; 3 School of Civil Engineering, Harbin Institute of Technology, Harbin, China; 4 School of Electrical Engineering and Automation, Harbin Institute of Technology, Harbin, Heilongjiang, China; Shandong University of Technology, CHINA

## Abstract

High strength and lightweight are key trends in concrete development. Achieving a balance between these properties to produce high structural efficiency (strength-to-weight ratio) concrete is challenging due to the complex relationship between compressive strength and material components. In this study, two artificial neural network (ANN) models-the BP and Elman networks were used to predict the compressive strength of ultra-high-performance lightweight concrete (UHPLC), based on a robust database of 115 test datasets from previous studies. The investigated parameters included the cement grade (Grade 42.5 and Grade 52.5), cement content (352 kg/m^3^-938 kg/m^3^), silica fume content (0 kg/m^3^-350 kg/m^3^), fly ash content (0 kg/m^3^-220 kg/m^3^), microsphere content (0 kg/m^3^-624 kg/m^3^), lightweight sand types (pottery sand, expanded perlite sand, and expanded shale lightweight sand), lightweight sand content (0 kg/m^3^-769 kg/m^3^), sand type (quartz sand, river sand), sand content (0 kg/m^3^-1314 kg/m^3^), water (90 kg/m^3^-395 kg/m^3^), water reduce (0 kg/m^3^-42.8 kg/m^3^), steel fiber content (0 kg/m^3^-234 kg/m^3^). Correlation analysis and sensitive analysis indicated that lightweight sand content and sand content had the most significant effects on UHPLC compressive strength, followed by water content. Conversely, fly ash content and lightweight sand type had minimal impact. The developed ANN models for UHPLC compressive strength demonstrated high predictive accuracy for both training and testing datasets, which the RMSE of BP network and Elman network were 0.226 and 0.160, respectively, while R^2^ of both two developed models were more than 0.98. Additionally, UHPLC exhibited a higher compressive strength-to-density ratio than high-strength concrete, ultra-high-performance concrete, and even Q235 steel. Three strategies were proposed for creating ultra-high-performance lightweight composites: optimizing packing density and lowering the water-binder ratio, along with careful selection of lightweight aggregates.

## 1. Introduction

The development of concrete is trending towards achieving both high strength and light weight. However, it is still a significant challenge to balance the relationships between high strength and light weight to create high structural efficiency (strength-to-weight ratio) concrete. High strength and light weight are often seen as two opposing characteristics, and the compressive strength of concrete decreases as its weight is reduced. Yet, in recent years, with the increasing demands for super high-rise buildings, long-span bridges, and offshore platforms, the practical need for high-strength lightweight concrete has grown. In addition, for modular integrated construction, high-strength lightweight concrete offers advantages in lifting efficiency, lower transportation costs, and improved sound insulation. Beyond being lightweight and high-strength, high-strength lightweight concrete also offers other benefits over conventional concrete, including enhanced thermal insulation, better durability, and cost savings [1,2].

Ultra-high performance lightweight concrete (UHPLC) is a novel cement-based material that combines high strength, exceptional durability, and light weight. The preparation principle of UHPLC is based on the preparation method of ultra-high performance concrete by introducing lightweight aggregates, (such as pumice, scoria expanded clay, shale, et al) to decrease of weight of concrete. The compressive strength of UHPLC exceeds 60MPa, and the density of UHPLC was below 1980 kg/m^3^. However, it is remains a huge challenge to accurately predict the compressive strength of UHPLC due to the variability of cement-based materials, and the differences in the type, density and strength of lightweight aggregate [3–5].

Many researchers have investigated in the mixture of UHPLC. Wang et al. replaced quartz sands with cenosphere, achieving UHPLC with an apparent density below 1815 kg/m^3^, compressive strength exceeding 93.51 MPa, and tensile strength less than 7.6 MPa under standard curing conditions [6]. Ding et al. studied the effects of gel composition, binder-to-sand ratio, and steel fiber content on the workability and mechanical properties of UHPC to obtain the optimal mixtures of UHPC [7]. Zhang et al. observed that a 13% prewater absorption rate of clay ceramisite can maximize the compressive strength and tensile strength of UHPC. With the increase in the content of lightweight aggregates, the fluidity and compressive strength of UHPLC showed a trend of first increasing and then decreasing. A UHPLC with an apparent density of 2040 kg/m^3^ and the compressive strength over 110 MPa was achieved using this mix design [8]. Xiang found that with the increase of lightweight aggregates size, the fluidity and mechanical properties of UHPLC were improved [9]. Pan et al. examined that the effects of the content of hollow glass microsphere on the mechanical properties of UHPLC. The results showed that with the increase in the content of hollow glass microsphere, the apparent density and mechanical properties of UHPLC decreased [10]. Cakir et al. investigated that the effects of curing regime and pre-mechanical pressure on the density, water absorption and compressive strength of UHPLC [11]. Meng et al. found that a 25% replacement rate of lightweight sand resulted in UHPC with compressive strength and flexural strength of 168 MPa and 24 MPa, respectively [12]. Based on the previous studies, researchers only focused on the effects of water-to-binder ratio, lightweight aggregates types and content on the workability and mechanical properties of UHPLC. Compressive strength is an important index to measure the mechanical properties of UHPLC. Previous studies have demonstrated that the compressive strength of UHPLC was affected by the factors, such as the composition and content of cementitious materials, the type, content, and prewater absorption rate of lightweight aggregates, and fiber content. However, a predictive formula for evaluating the compressive strength of UHPLC that considered the above influential parameters was currently lacking. Thus, it was necessary to develop a reliable method for evaluating the compressive strength of UHPLC.

In recent years, artificial intelligence techniques, particularly artificial neural networks (ANNs), have been widely used to address complex engineering problems due to their high accuracy and adaptability. The ANN model has been successfully applied to evaluate early-age autogenous shrinkage of concrete [13], estimate the compressive strength of FRP-confined concrete circular columns [14], optimize the mix compositions of steel fiber-reinforced concrete [15], evaluate the elastic modulus [16] and mixtures [17] of concrete, analyze the compressive strength of stirrup-confined concrete columns [18,19], estimate of geopolymer concrete compressive strength [20], and so on. However, few studies have been conducted on applying ANNs to predict the compressive strength of UHPLC. As for predicting the effects of material properties on the compressive strength of UHPLC, because of high adaptability and high accuracy, the ANN models are suitable for evaluating the mechanical behaviour of UHPLC.

The aim of this study is to develop two ANN models to predict the compressive strength of UHPLC. To achieve this purpose, 115 datasets of compressive strength were collected from the published literature, allowing for model development and analysis of materials properties effects on the compressive strength of UHPLC. Finally, the proposed models were compared with several analytical models to assess their reliability and predictive accuracy. Additionally, the strategies for preparing UHPLC with high compressive strength were proposed.

## 2. Artificial neural network approach

### 2.1. BP network introduction

This article does not contain any studies with animals performed by any of the authors.

Artificial neural network (ANN) is an advanced approach for addressing complex engineering problems due to its high adaptability and strong predictive capabilities. A back propagation (BP) neural network, as a type of multi-layer feed-forward neural network, is particularly effective for solving nonlinear problems. The BP network uses a supervised learning method to train the model by minimizing the errors between calculated and experimental values, adjusting the weights incrementally with each iteration [21,22].

To enable accurate predictions, the BP network should undergo a training process, which includes five stages [21,22]: network initialization, hidden layer calculation, output layer calculation, errors calculation, and weight update. The structure of the BP network, including the number of nodes in the input and output layers, is determined based on the specific research questions. Initial weights and thresholds are assigned to the hidden layers to initiate the training process. The hidden layer outputs are then generated by passing input layer values through the hidden layers, with weights and thresholds between these layer calculated using activation functions. A common choice for the activation function is the Sigmoid function, as expressed in Eq 1.


$$f(x)=\frac{1}{1+e^{-x}}$$ (1)

The output layer values are computed by transferring the hidden layer outputs to the output layer. The discrepancies between predicted and target values are then calculated to adjust the weights and thresholds between the hidden and output layers. Once training is complete, the BP model stabilizes, with the weights and thresholds converging to consistent values.

The Levenberg-Marquardt (LM) algorithm is a widely used training method within BP networks due to its rapid convergence and high accuracy compared to other backpropagation techniques. The LM algorithm is particularly suitable for nonlinear least squares problems and curve fitting, as it combines the benefits of the Quasi-Newton method and Steepest Descent Backpropagation.

### 2.2. Elman network introduction

The Elman network is a dynamic recurrent neural network based on the BP network architecture. The Elman network includes an additional layer known as the context layer, where context neurons retain a copy of the output from the hidden layer neurons. In each subsequent computational step, this stored information is provided as new input to the context layer, enabling the network to process temporal information and retain previous states [23–25].

In an Elman network, the connections between neurons are defined by their weight values, which are initially assigned randomly. During model training, these weights are adjusted to optimize performance, with the exception of the weights linking the hidden layer to the context layer. These remain unchanged throughout the training process to ensure that the context neurons consistently receive the exact output values from the hidden layer. The Elman network was trained using the Levenberg-Marquardt (LM) algorithm.

## 3. Database preparation

### 3.1. Database

To establish the suitable ANN models for predicting the compressive strength UHPLC, a database comprising 115 datasets was assembled from previous studies, as detailed in Table 1. The investigated parameters included the cement grade (CG), cement content (C), silica fume content (SF), fly ash content (FA), microsphere content (MC), lightweight sand types (LWST), lightweight sand content (LWSC), sand type (ST) (Quartz sand (QS) and river sand (RS)), sand content (SC), water content (W), water reducer content (WR), and steel fiber content (STF).

**Table 1:** Experimental Data used for establishing compressive strength ANN model.

CG	C	SF	FA	MC	LWST	LWSC	ST	SC	W	WR	STF	fcu/MPa	Ref.
52.5	938	108	0	256	PS	0	QS	121	233	3.2	156	93.5	[6]
52.5	938	108	0	256	PS	0	QS	121	233	3.2	156	103.1	
52.5	938	108	0	256	PS	0	QS	121	233	3.2	156	96.8	
52.5	938	108	0	256	PS	0	QS	121	233	3.2	156	98.6	
42.5	800	150	0	200	PS	0	QS	1045	207	0.9	156	135.0	[26]
42.5	800	150	0	200	PS	30	QS	993	207	0.9	156	128.4	
42.5	800	150	0	200	PS	60	QS	941	207	0.9	156	121.3	
42.5	800	150	0	200	PS	89	QS	888	207	0.9	156	116.0	
42.5	800	150	0	200	PS	119	QS	836	207	0.9	156	112.3	
42.5	800	150	0	200	PS	149	QS	783	207	0.9	156	105.0	
42.5	800	150	0	200	PS	179	QS	731	207	0.9	156	101.6	
42.5	800	150	0	200	PS	209	QS	697	207	0.9	156	92.3	
42.5	800	150	0	200	PS	238	QS	627	207	0.9	156	87.0	
42.5	800	150	0	200	PS	268	QS	575	207	0.9	156	81.0	
42.5	800	150	0	200	PS	298	QS	523	207	0.9	156	75.8	
42.5	800	350	0	0	PS	0	QS	1045	207	0.9	156	112	
42.5	800	300	0	50	PS	0	QS	1045	207	0.9	156	115	
42.5	800	250	0	100	PS	0	QS	1045	207	0.9	156	126.5	
42.5	800	200	0	150	PS	0	QS	1045	207	0.9	156	135.0	
42.5	800	150	0	200	PS	0	QS	1045	207	0.9	156	130.0	
42.5	800	100	0	250	PS	0	QS	1045	207	0.9	156	123.0	
42.5	800	50	0	300	PS	0	QS	1045	207	0.9	156	112.5	
42.5	800	0	0	350	PS	0	QS	1045	207	0.9	156	108.0	
52.5	810	200	0	190	PS	667	QS	0	204	34	0	72.8	[27]
52.5	810	200	0	190	PS	667	QS	0	204	34	78	97.2	
52.5	810	200	0	190	PS	667	QS	0	204	34	117	106.9	
52.5	810	200	0	190	PS	667	QS	0	204	34	156	114.2	
52.5	810	200	0	190	PS	667	QS	0	204	34	195	121.2	
42.5	650	150	0	100	PS	0	RS	1300	153	8.5	80	120.7	[28]
42.5	650	150	0	100	PS	300	RS	600	153	8.5	80	68.8	
42.5	650	150	0	100	PS	326	RS	600	124	8.5	80	75.0	
42.5	650	150	0	100	PS	300	RS	760	153	8.5	80	93.6	
42.5	650	150	0	100	PS	312	RS	760	140	8.5	80	99.0	
52.5	721	185	0	123	ES	596	QS	0	181.2	2.0	156	68.5	[9]
52.5	710	185	0	134	ES	596	QS	0	179.0	2.0	156	72.3	
52.5	700	185	0	144	ES	596	QS	0	177.0	2.0	156	73.5	
52.5	690	185	0	154	ES	596	QS	0	175.0	2.0	156	78.6	
52.5	679	185	0	165	ES	596	QS	0	172.8	2.0	156	71.9	
52.5	710	165	0	154	ES	596	QS	0	175.0	2.0	156	72.1	
52.5	700	175	0	154	ES	596	QS	0	175.0	2.0	156	75.8	
52.5	690	185	0	154	ES	596	QS	0	175.0	2.0	156	80.9	
52.5	679	196	0	154	ES	596	QS	0	175.0	2.0	156	86.9	
52.5	669	206	0	154	ES	596	QS	0	175.0	2.0	156	78.8	
52.5	679	196	0	154	ES	596	QS	0	148.8	2.0	156	94.5	
52.5	679	196	0	154	ES	596	QS	0	157.5	2.0	156	93.8	
52.5	679	196	0	154	ES	596	QS	0	166.2	2.0	156	89.4	
52.5	679	196	0	154	ES	596	QS	0	175	2.0	156	86.5	
52.5	679	196	0	154	ES	596	QS	0	157.5	2.0	156	90.2	
52.5	679	196	0	154	ES	596	QS	0	157.5	2.0	156	93.7	
52.5	679	196	0	154	ES	626	QS	0	157.5	2.0	156	89.3	
52.5	679	196	0	154	ES	626	QS	0	157.5	2.0	156	82.5	
52.5	679	196	0	154	ES	596	QS	0	157.5	2.0	78	84.7	
52.5	679	196	0	154	ES	596	QS	0	157.5	2.0	117	88.7	
52.5	679	196	0	154	ES	596	QS	0	157.5	2.0	156	93.7	
52.5	679	196	0	154	ES	596	QS	0	157.5	2.0	195	101.6	
52.5	679	196	0	154	ES	596	QS	0	157.5	2.0	234	108.8	
52.5	781	187	198	0	PS	0	QS	0	209.9	42.8	218	126.5	[29]
52.5	737	176	187	0	PS	0	QS	0	198	41.4	218	125.1	
52.5	781	187	198	0	ES	480	QS	0	209.9	32.9	218	113.5	
52.5	7.7	176	187	0	ES	480	QS	0	198	31.6	218	110.1	
52.5	781	187	198	0	PS	480	QS	0	209.9	32.9	218	120.9	
52.5	737	176	187	0	PS	480	QS	0	198	31.6	218	118.2	
52.5	737	176	187	0	PS	480	QS	0	198	31.6	218	109.8	
52.5	737	176	187	0	PS	480	QS	0	198	31.6	218	103.5	
52.5	781	187	198	0	PS	480	QS	0	209.9	32.9	218	105.6	
52.5	840	168	0	192	ES	706	QS	0	151.2	0	156	94.8	[30]
52.5	828	168	0	204	ES	706	QS	0	149.0	0	156	100.6	
52.5	804	168	0	228	ES	706	QS	0	144.7	0	156	101.2	
52.5	804	192	0	204	ES	706	QS	0	144.7	0	156	110.5	
52.5	804	204	0	192	ES	706	QS	0	144.7	0	156	112.9	
52.5	429	188	0	0	EPS	302	RS	845	177	30	0	130.2	[31]
52.5	429	188	0	0	EPS	592	RS	145	363	30	0	72.5	
52.5	803	188	0	0	EPS	397	RS	145	371	30	0	100.3	
52.5	803	188	0	0	EPS	107	RS	845	182	30	0	120.0	
52.5	352	188	0	0	EPS	503	RS	495	325	30	0	88.4	
52.5	880	188	0	0	EPS	226	RS	495	287	30	0	99.6	
52.5	616	188	0	0	EPS	560	RS	0	395	30	0	90.1	
52.5	616	188	0	0	EPS	138	RS	990	174	30	0	135.2	
52.5	616	188	0	0	EPS	364	RS	495	276	30	0	110.9	
52.5	616	188	0	0	EPS	364	RS	495	283	30	0	108.2	
52.5	616	188	0	0	EPS	364	RS	495	280	30	0	110.7	
52.5	429	188	0	0	EPS	302	RS	845	177	30	156	167.3	
52.5	429	188	0	0	EPS	592	RS	145	363	30	156	122.0	
52.5	803	188	0	0	EPS	397	RS	145	371	30	156	127.1	
52.5	803	188	0	0	EPS	107	RS	845	182	30	156	188.1	
52.5	352	188	0	0	EPS	503	RS	495	252	30	156	121.9	
52.5	880	188	0	0	EPS	226	RS	495	287	30	156	132.7	
52.5	616	188	0	0	EPS	560	RS	0	395	30	156	123.8	
52.5	616	188	0	0	EPS	138	RS	990	174	30	156	186.6	
52.5	616	188	0	0	EPS	364	RS	495	276	30	156	134.2	
52.5	616	188	0	0	EPS	364	RS	495	283	30	156	140.1	
52.5	616	188	0	0	EPS	364	RS	495	280	30	156	134.4	
52.5	894	89	179	0	ES	0	RS	930	231	12	66	117.3	[32]
52.5	741	74	148	0	ES	0	RS	1155	222	6	55	113.3	
52.5	632	63	126	0	ES	0	RS	1315	214	3	47	84.8	
52.5	551	55	110	0	ES	0	RS	1434	207	2	41	82.6	
52.5	890	89	178	0	ES	499	RS	0	146	35	66	114.1	
52.5	739	74	148	0	ES	620	RS	0	121	29	55	82.6	
52.5	631	63	126	0	ES	705	RS	0	103	25	47	72.2	
52.5	551	55	110	0	ES	769	RS	0	90	21	41	71.1	
52.5	650	150	200	208	ES	490	RS	0	180	20	156	99.6	[33]
52.5	650	150	200	277	ES	435	RS	0	180	20	156	102.4	
52.5	650	150	200	415	ES	325	RS	0	180	20	156	105.5	
52.5	650	150	200	555	ES	217	RS	0	180	20	156	103.4	
52.5	650	150	200	624	ES	163	RS	0	180	20	156	99.5	
52.5	650	150	200	415	ES	325	RS	0	180	20	156	105.5	
52.5	666	154	205	407	ES	319	RS	0	185	20	156	108.2	
52.5	685	158	210	398	ES	312	RS	0	189	20	156	114.5	
52.5	699	161	215	389	ES	305	RS	0	194	20	156	96.1	
52.5	715	165	220	379	ES	297	RS	0	198	20	156	91.1	
52.5	650	150	200	426	ES	334	RS	0	180	20	39	73.1	
52.5	650	150	200	421	ES	330	RS	0	180	20	78	85.4	
52.5	650	150	200	415	ES	325	RS	0	180	20	117	93.7	
52.5	650	150	200	415	ES	325	RS	0	180	20	156	105.5	
52.5	650	150	200	407	ES	319	RS	0	180	20	195	94.5	

Note: CG, C, SF, FA, MC, LWST, LWSC, ST, QS, RS, SC, W, WR, and STF were denoted as the cement grade, cement content, silica fume content, fly ash content, microsphere content, lightweight sand types, lightweight sand content, sand type, quartz sand, river sand, sand content, water content, water reducer content, and steel fiber content, respectively, Fcu was denoted as the compressive strength of UHPLC. As for LWST, in the established ANN models, the PS, ES, EPS were denoted as 1,2,3, respectively; as for ST, the QS and RS were denoted as 1, 2, respectively.

To ensure the reliability of the established database, the following criteria were applied: (1) the concrete compressive strength values corresponded to cubic compressive strength, which the dimension of cube was 100 mm × 100 mm × 100 mm; (2) the microsphere type used was fly ash microsphere (FAM); (3) lightweight sand types included pottery sand (PS), expanded perlite sand (EPS), and expanded shale lightweight sand (ES); (4) the particle size range of lightweight sand across different studies was sufficiently similar, so variations in particle size were not considered; (5) the UHPLC was cured under standard curing regime, with the curing temperature of 20°C ~ 25°C and the curing regime humidity of 95%; (6) the compressive strength of UHPLC was obtained by curing 28 days.

**Fig 1:**
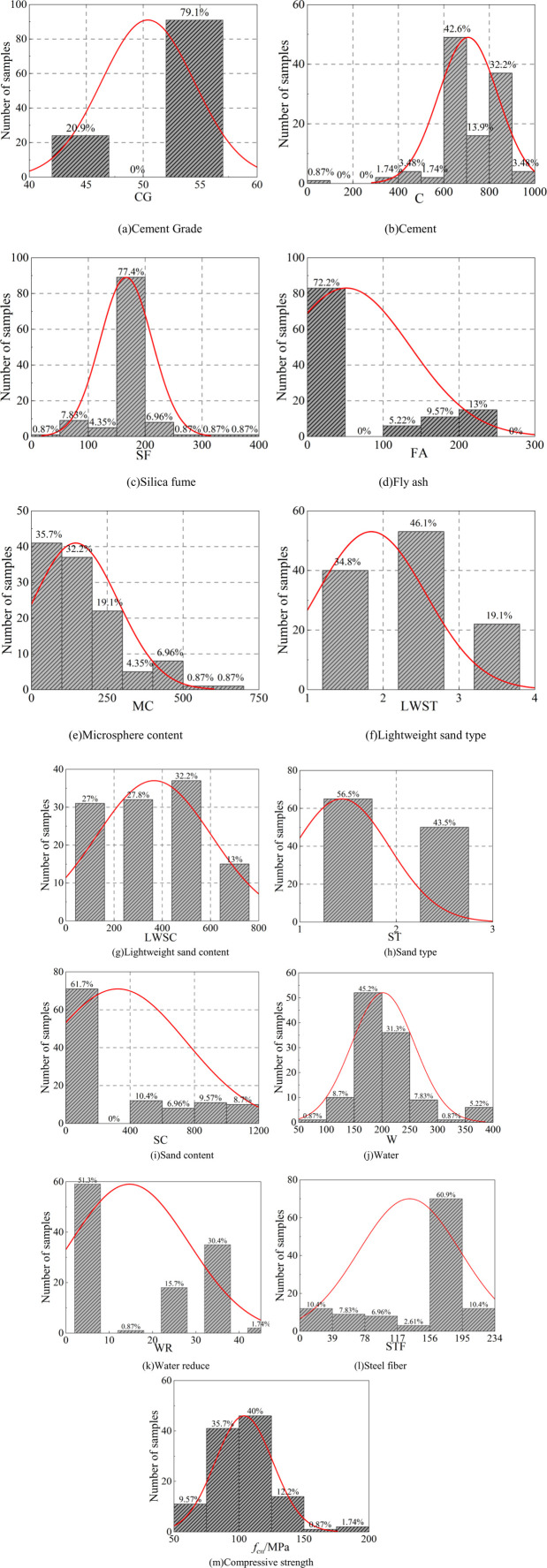
Distributions of collected data sets.

The distribution of collected data sets is shown in Fig 1, and the specific ranges of the investigated parameters are listed in Table 2.

**Table 2:** Specific ranges of investigated parameters.

Ranges	CG	C	SF	FA	MC	LWST	LWSC	ST	SC	W	WR	STF	fcu/MPa
Min. value	42.5	352	0	0	0	_	0	_	0	90	0	0	68.5
Max. value	52.5	938	350	220	624	_	769	_	1434	395	42.8	234	188.1
Median value	52.5	700	185	0	154	_	364	_	0	198	8.5	156	102.4

**Fig 2:**
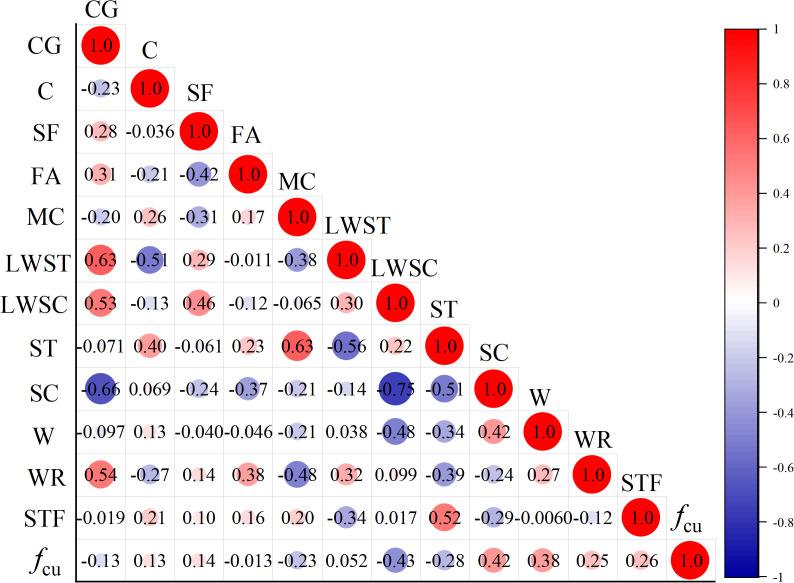
Correlation analysis among parameters.

Fig 2 is the correlation analysis among parameters. As shown in Fig 2, lightweight sand content and sand content significantly affected the compressive strength of UHPLC, followed by water content. In contrast, fly ash content and lightweight sand type had minimal impact on UHPLC compressive strength.

Therefore, to develop ANN models for predicting the compressive strength of UHPLC, the primary influencing parameters considered were cement grade, cement content, silica fume content, microsphere content, lightweight sand content, sand type, sand content, water content, water reducer content, and steel fiber content.

### 3.2. Model establishments

In this study, typical ANN models (including the BP network and Elman network) were chosen to evaluate the compressive strength of UHPLC, established by Python software. The ANN model demonstrating the highest predictive performance was selected. To develop these models, 80% of the datasets were randomly assigned as training sets, with the remaining 20% used for testing. Random selection of testing data minimized potential biases from manual selection, ensuring more reliable results.

All networks developed in this study to evaluate the compressive strength of UHPLC consisted of three layers: an input layer, a single hidden layer, and an output layer. The parameters used in each proposed network for UHPLC compressive strength prediction are listed in Table 3. To determine the nodes nomber in the hidden layers, the nodes number of hidden layers for BP network ranging from 10 to 20 and the nodes number of hidden layers for Elman network ranging from 10 to 25 were carried out. The root mean square error (RMSE), an absolute fraction of variance ($R^{2}$) were carried out to evaluate the prediction performance of established ANN model. Finally, the nodes number of hidden layers for BP network and Elman network were determined as 15 and 20, respectively.

**Table 3:** Parameters of proposed models for peak stress of concrete columns.

Parameters	BP network	Elman network
Number of input layer nodes	10	10
Number of hidden layers	1	1
Number of hidden layer nodes	15	20
Number of output layer nodes	1	1
Training algorithms	Levenberg-Marquardt	Levenberg-Marquardt
Gradient	10^−7^	10^−7^
Activated fuction	Sigmoid	Tahn
Momentum factor	0.8	0.8
Learning rate	0.3	0.3
Target error	0.00001	0.00001

In all networks, the collected data was normalized within the specific limits before training and testing. Normalization of data can eliminate the non-singular data, improve the precision of results, accelerate the convergence speed, and reduce the calculation time. A sample function expressed in Eq 2 was adopted to normalize the data in this study.


Xi,norm=0.1+0.8×(Xi−Xmin)/(Xi−Xmin)(Xmax−Xmin)\nulldelimiterspace(Xmax−Xmin) (2)

**Fig 3:**
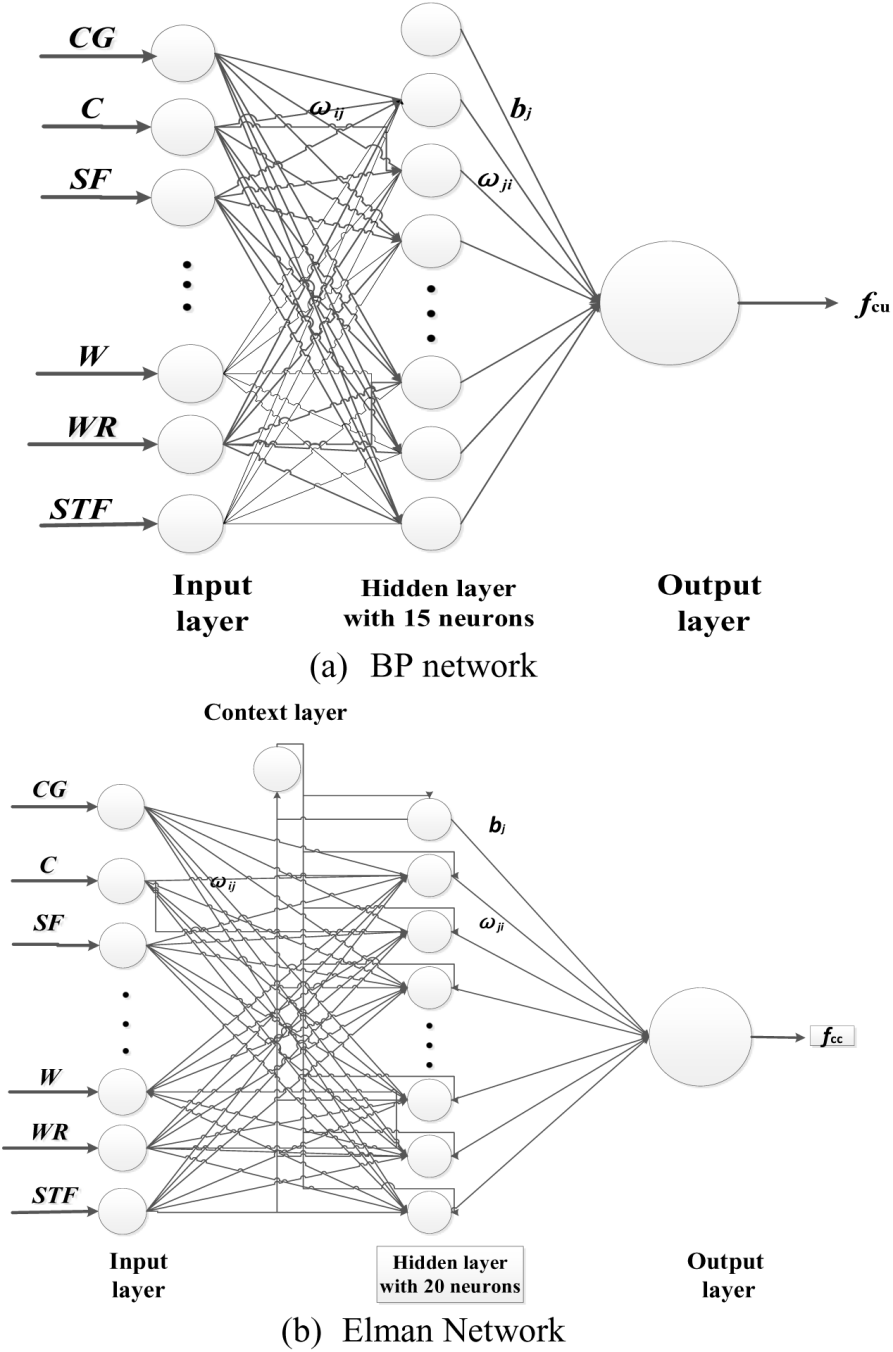
Structures of established BP and Elman networks for compressive strength of UHPLC.

Where $X_{i,norm}$ is normalized data,$X_{\max}$ and$X_{\min}$ are the maximum and minimum values of data, respectively. An inverse normalized process is applied to the output layer to get the test data. The structures of the established BP network and Elman network are shown in Fig 3.

## 4. Results and discussion

### 4.1. Prediction results evaluation

**Fig 4:**
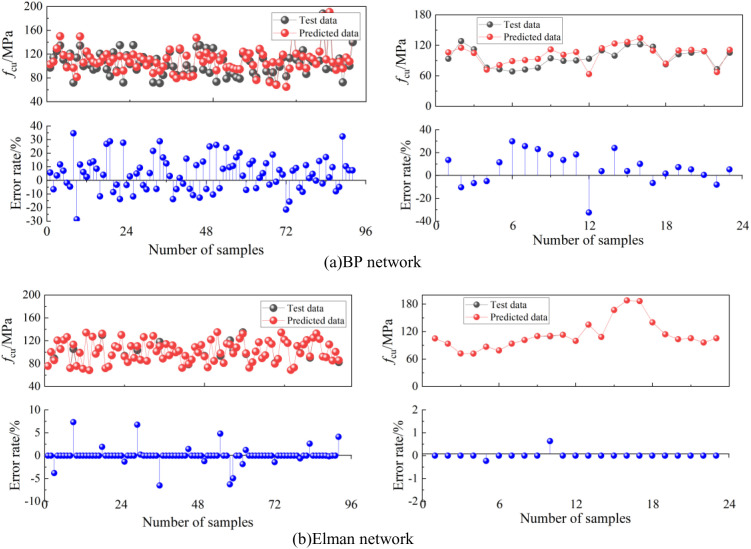
Training and testing process of proposed ANN models for ductility coefficient.

Fig 4 shows the comparison between predicted and experimental results for the training and testing datasets in the proposed ANN models for UHPLC compressive strength. The predicted values from both the BP network and Elman network closely matched the target values, with minimal error between predicted and actual values. This indicates that both networks successfully captured the nonlinear relationship between input and output variables, demonstrating their potential for accurately evaluating the compressive strength of UHPLC.

### 4.2. Evaluation prediction performance of proposed models

A successful prediction model should give an accurate output prediction. In this study, three indicators were applied to evaluate the prediction performance of the proposed models, which included the root mean square error (RMSE), an absolute fraction of variance ($R^{2}$), and α20−index, which was expressed in Eqs 3–Eq 5.


$$RMSE=\sqrt{\frac{1}{n}\sum_{k=1}^{n}{\left(t_{k}-o_{k}\right)}^{2}}$$ (3)

R2=1−[∑i=1n(ti−oi)2/∑i=1n(ti−oi)2∑i=1nti2\nulldelimiterspace∑i=1nti2] (4)

$$\alpha 20-index=1-\frac{n_{20}}{n}$$ (5)

Wheren was the total number of samples; $t_{k}$ was the experimental result of $k^{th}$ data; $o_{k}$ was the predicted results of $k^{th}$ data; $n_{20}$ was the number of samples in which the values of the ok/oktk\nulldelimiterspacetk were in the range of 0.8–1.2.

**Fig 5:**
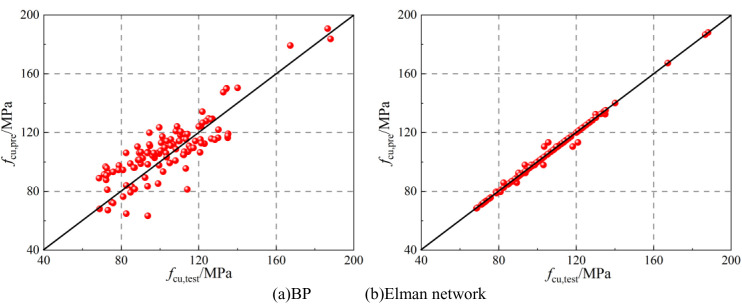
Comparison between predicted results and test results.

**Fig 6:**
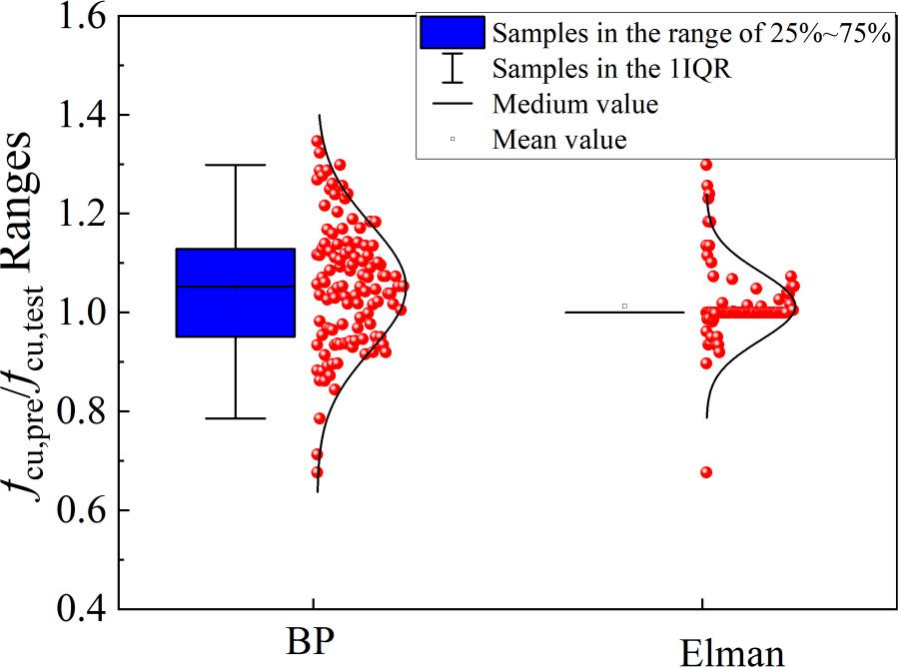
Box plot of *f*_cu,pre_/*f*_cu,test_ with different prediction models.

The value of R^2^ was applied to evaluate the variation between predicted results and experimental results. The value of RMSE was applied to evaluate the errors between predicted results and experimental results. The value of α20−index was the ratio between predicted results and actual values out of the range of 0.8–1.2. Theoretically, the higher R^2^ and lowest RMSE indicated the good prediction performance of proposed models, while α20−index was expected to be 0 in the perfect prediction models. The values of three indicators for two proposed models are listed in Table 4. The comparison between predicted values and test results for two proposed models is shown in Fig 5, and the predicted values distributions is shown in Fig 6. As shown in Figs 5 and 6 and Table 4, it was highlighted that the proposed models were suitable for evaluating the compressive strength of UHPLC due to the lowest RMSE, and α20−index, and high R^2^.

**Table 4:** Three indicators for two proposed models.

Indicators	RMSE	R^2^	α20−index
BP network	6.225	0.986	0.157
Elman network	4.190	0.999	0

## 5. Sensitive analysis

**Fig 7:**
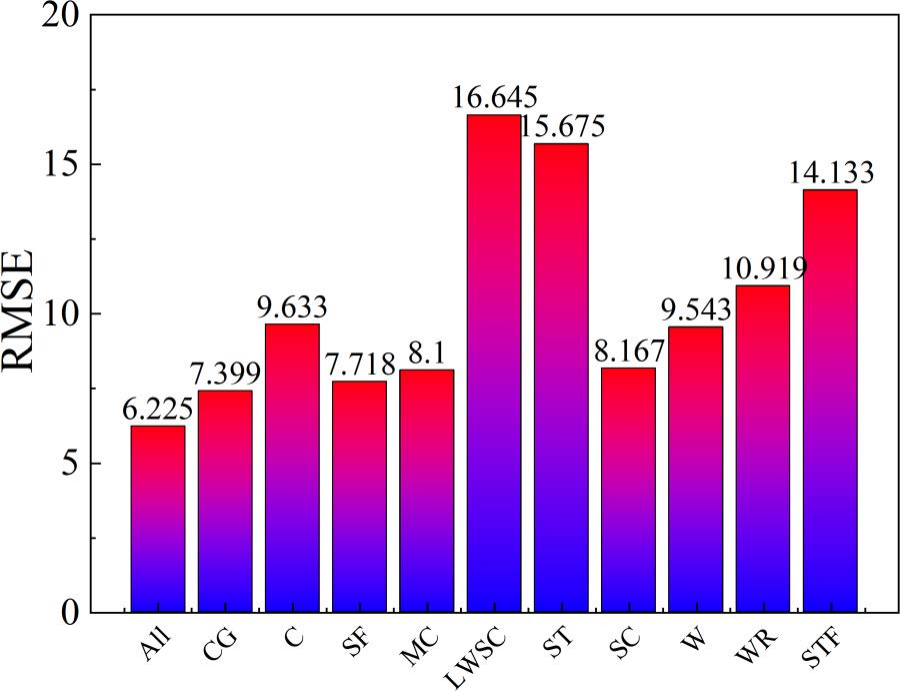
Parameter sensitive analysis.

Based on the established BP network, The parameters sensitive analysis was carried out and the performance indicators were calculated. Fig 7 shows that the evaluation of parameters sensitive in compressive strength model. From Fig 7, when the parameters mentioned in the paper were included for establishing the compressive strength model, the RMSE reached minimum. The sensitive of parameters was evaluated by excluding one certain parameters, and then established the prediction model was applied to calculate the RMSE. The RMSE reached maximum, the parameter excluded was important. From Fig 6, the lightweight sand content, sand type, and the steel fiber content were the top three parameters affects on the compressive strength of UHPLC.

## 6. Strategies for preparing UHPLC with high compressive strength

### 6.1. Ratio between compressive strength and density

**Fig 8:**
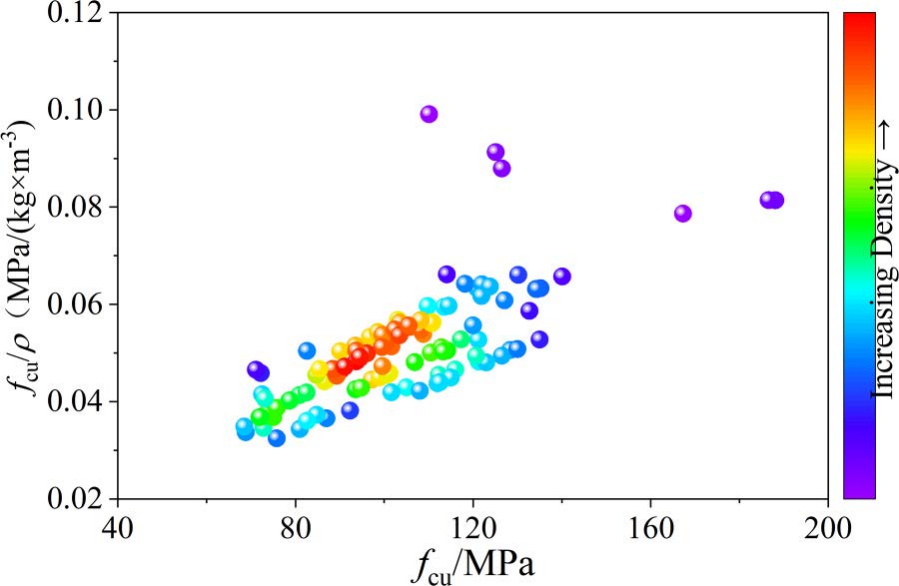
Range of the compressive strength/density ratio of UHPLC.

One of the key advantages of lightweight materials is the reduction in the dead load of structural components. The ratio of compressive strength to density (compressive strength/density) is commonly used to assess the material efficiency of lightweight concrete. Fig 8 illustrates the range of compressive strength/density ratios for UHPLC from the test results in the database (Table 1). As shown in Fig 8, the compressive strength/density ratio of UHPLC ranged from 0.032 MPa/(kg/m³) to 0.099 MPa/(kg/m³), surpassing that of high-strength concrete (0.025 MPa/(kg/m³)), ultra-high-performance concrete (0.05 MPa/(kg/m³)), and even Q235 steel (0.03 MPa/(kg/m³)). The compressive strength and density of UHPLC were primarily influenced by three factors: the paste matrix, lightweight materials, and their bonding quality. Based on these characteristics and UHPLC’s high specific strength, two strategies were proposed for developing ultra-high-performance lightweight composites.

### 6.2. Optimum packing density and low water-binder ratio

As shown in Fig 2, water content, cement content, and silica fume content were the primary factors affecting the compressive strength of concrete. Similar to ultra-high-performance concrete, an optimized packing density and low water-binder ratio promote a dense microstructure with minimal porosity, creating a robust matrix for UHPLC. In UHPLC, the cement paste binds lightweight particles, enhancing strength and providing resistance against harmful substance infiltration. Conventional lightweight concrete typically has lower mechanical properties and higher brittleness, limiting its applications. However, the addition of fibers has been shown to effectively increase tensile strength and toughness in lightweight concrete. UHPLC achieves high compressive strength by integrating an optimal blend of cementitious materials and steel fibers. From the established models, the water-to-bind ratio of UHPLC should not be more than 0.25.

### 6.3. Reasonable lightweight aggregates selection

As shown in Fig 2, lightweight aggregate significantly affects the compressive strength of UHPLC, with high-porosity aggregates acting as weak points in the material. Most lightweight aggregates have densities above 800 kg/m³, which prevents UHPLC from achieving very low densities. Therefore, optimizing the selection of lightweight aggregates is essential. The following principles should guide this selection: (1) High-strength, low-density aggregates-ideally under 800 kg/m³, such as hollow glass beads or fly ash beads; (2) Small particle size, as larger particles increase porosity and reduce strength; (3) Volcanic ash effect, where a hard shell forms on the aggregate’s surface to enhance UHPLC compressive strength.

## 7. Conclusions

In this paper, to evaluate the compressive strength of UHPLC, the artificial neural network (ANN) models, including the BP network and Elman network, were developed, and the effects of the main influential parameters on the compressive strength of UHPLC were evaluated. A reliable database of 115-group compressive strength test datasets from previous literature was applied to establish the prediction models. The conclusions were drawn as follows:

(1) Based on the correlation analysis, the lightweight sand content and sand content had obvious effects on the compressive strength of UHPLC, the water content was next. In contrast, the fly ash content and lightweight sand type had few effects on the compressive strength of UHPLC.
(2) The ANN models, including the BP network and Elman network for the compressive strength of UHPLC were developed, having high prediction performance for both training and testing data sets, which the RMSE of BP network and Elman network were 0.226 and 0.160, respectively, while R^2^ of both two developed models were more than 0.98. Thus, the proposed ANN models can be applied to the ranges of the parameters for the mixture design of UHPLC.
(3) UHPLC had a higher ratio between compressive strength and density than high-strength concrete, ultra-high performance concrete, and even Q235 steel. Three strategies including optimum packing density, low water-binder ratio, and reasonable lightweight aggregate selection to prepare ultra-high-performance lightweight composites were proposed.
(4) Research gaps: 1) the established models did not consider the effects of the partical size distribution of lightweight aggregates; 2) the effects of fibers which the fiber types were more than two types on the compressive strength of UHPLC were not considered; 3) the artificial intelligent design platform for the mixture design of UHPLC should do further studies.
